# Decreased complement 4 and interleukin-10 as biomarkers in aqueous humour for non-exudative age-related macular degeneration: a case control study

**DOI:** 10.1186/s12967-024-05909-x

**Published:** 2025-03-12

**Authors:** Juliane Schikora, Aaron Dort, Hannah N. Wolf, Mihály Józsi, Richard B. Pouw, Thomas Bertelmann, Dirk Bahlmann, Christian van Oterendorp, Nicolas Feltgen, Hans Hoerauf, Diana Pauly, Jannis Klemming

**Affiliations:** 1 Experimental Ophthalmology, University Marburg, Marburg, Germany; 2 Department of Immunology, ELTE Eötvös Loránd University, Budapest, Hungary; 3 Sanquin Research and Landsteiner Laboratory, Amsterdam University Medical Centers, University of Amsterdam, Amsterdam, The Netherlands; 4 Department of Ophthalmology, University Medical Center Göttingen, Göttingen, Germany; 5 Department of Ophthalmology, University Basel, Basel, Switzerland

**Keywords:** Aqueous fluid, Biomarker, Chemokine, Complement regulation, Cytokine, Drusen, Geographic atrophy, Interleukin, Retina, Smoking, Sex

## Abstract

**Background:**

The development of age-related macular degeneration (AMD) is influenced by risk factors that contribute to inflammatory processes, cellular stress responses, and a dysregulation of the complement system. Given the incomplete understanding of the pathogenesis of AMD and the necessity for novel therapeutics, biomarker studies investigating aqueous humour from the anterior chamber of the eye serve as a valuable tool. This pilot study aimed to assess inflammatory mediators and complement components in aqueous humour of non-exudative AMD patients in comparison with a control group.

**Methods:**

The aqueous humour of 12 non-exudative AMD patients and 21 control subjects was collected during cataract surgery. Levels of 78 inflammatory proteins and complement components were measured using multiplex immunoassays. The influence of sex or smoking on the AMD status was assessed using Pearson’s chi-square test. Biomarker levels between AMD patients vs. controls, smokers vs. non-smokers, and females vs. males were compared. Parametric datasets were analysed using independent-means *t*-test, while non-parametric data analysis was conducted utilising Wilcoxon’s rank-sum test. Spearman’s correlation investigated associations between drusen volume and biomarker levels, as well as biomarker levels and subject age.

**Results:**

All examined 78 immunological factors were detectable in aqueous humour. The proteins were categorised into high, medium, and low level groups. Aqueous humour contained high levels of complement proteins, including iC3b, FH/FHL-1, C4B, and FI. Non-exudative AMD patients exhibited decreased levels of C4 (*P* = 0.020), IL-10 (*P* = 0.033), and FI (*P* = 0.082). A positive correlation was observed between drusen volume and CCL4 levels (*r*_*S*_ = 0.78, *P* = 0.013). Furthermore, smokers demonstrated significantly increased levels of pro-inflammatory proteins (CCL7, IL-7; *P* = 0.027, *P* = 0.030). MMP-1 was positively correlated with age (*r*_*S*_ = 0.44, *P* = 0.010), while sex differences were observed in FB (*P* = 0.027) and C4B (*P* = 0.036) levels.

**Conclusions:**

This pilot study presents an initial overview of inflammation-associated biomarkers in the aqueous humour, highlighting potential roles for C4 and IL-10 in the development of non-exudative AMD. A larger, more-focused follow-up study is in progress to further investigate biomarkers localised to the eye and refine our understanding of AMD.

**Supplementary Information:**

The online version contains supplementary material available at 10.1186/s12967-024-05909-x.

## Background

Age-related macular degeneration (AMD) stands as a significant public health concern, particularly in high-income countries, due to its emergence as the leading cause of blindness in the elderly population [1, 2]. It is characterised by progressive damage to the macula, leading to loss of central vision. AMD is broadly classified into two major subtypes: exudative (wet, neovascular) AMD and non-exudative (dry, non-neovascular, geographic atrophy) AMD [3]. While therapeutic advancements with vascular endothelial growth factor (VEGF) inhibitors have revolutionised the treatment landscape for exudative AMD, only limited therapeutic options exist for the non-exudative AMD variant [4, 5].

AMD is a complex disease with a pathogenesis that has not yet been fully understood. Its development is influenced by various risk factors, including ageing, smoking, and photo-oxidative stress. These factors contribute to cellular stress responses, which subsequently induce inflammasome activation and enhanced secretion of inflammatory mediators, such as tumour necrosis factor alpha (TNF-α) [6, 7]. In response, retinal cells, particularly retinal pigment epithelium (RPE) cells, increase the production of cytokines, chemokines, and regulatory molecules, including interleukin-6 (IL-6), interleukin-18 (IL-18), CC-chemokine ligand 7 (CCL7), and matrix metalloproteinase-1 (MMP-1) [6, 8–11]. These proteins exhibit diverse pro- and anti-inflammatory functions, impairing the functionality of RPE cells, recruiting immune cells to the retina and maintaining a persistent state of chronic inflammation that further exacerbates the development and progression of AMD [12, 13].

In addition to cellular stressors, the complement system, as an element of the innate immune response, significantly contributes to the pathogenesis of AMD. In general, the complement system is activated through multiple pathways, facilitating the removal of pathogens and cellular debris [14]. A key initiating factor of the classical and lectin pathways is the cleavage of complement 4 (C4), producing complement 4a (C4a) and 4b (C4b), which subsequently form complement 3 (C3) convertases, promoting further C3 activation. This cascade progresses to the terminal pathway, resulting in the formation of the membrane attack complex [15, 16]. The activation of the complement cascade is tightly regulated by several factors. Complement factor I (FI) and its co-factors, e.g. complement factor H (FH), inhibit the activation product of C3 and attenuate inflammatory effects [17]. Genome wide association studies have identified numerous single nucleotide polymorphisms or variants within complement genes, such as *C4*, complement factor H (*CFH*), and complement factor I (*CFI*), that significantly influence AMD susceptibility [18–27]. Altered complement function and regulation attract immune cells to the retina, induce pro-inflammatory secretion from RPE cells and photoreceptors, activate the inflammasome and finally augment AMD pathogenesis [9, 28–30]. During the development and progression of AMD, characteristic drusen deposits form between the RPE and Bruch's membrane. Previous studies have demonstrated that, in addition to lipid and waste accumulations, drusen contain complement components, such as complement 9 (C9) and FH [31–34]. Notably, the Food and Drug Administration (USA) has recently approved two agents (Avacincaptad pegol and Pegcetacoplan) targeting the complement system to treat patients with geographic atrophy, highlighting the increasing recognition of the importance of the complement pathway [35–37].

To find new therapeutic options for AMD, research has focused on identifying biomarkers in the aqueous humour (AH) of the eye’s anterior chamber. Studies have aimed to compare inflammatory proteins in the AH of non-exudative AMD patients with those of control subjects. In general, a multitude of inflammatory mediators have been identified in the AH, which includes IL-6, interleukin-7 (IL-7), interleukin-10 (IL-10), interleukin-16 (IL-16), CCL7, CC-chemokine ligand 20 (CCL20), fibroblast growth factor 2 (FGF-2), and TNF-α, as well as complement components like C4, complement factor B (FB), FH, and FI [38–42]. However, no significant differences in the levels of these proteins were found in the AH of AMD patients compared to controls.

This pilot study aimed to further address the understanding of the role of the immune system in the pathophysiology of non-exudative AMD by identifying initial biomarker targets for future investigations. First, we comprehensively analysed and categorised the presence of 78 inflammatory mediators and complement components in the AH. Second, we compared the levels of inflammatory mediators and complement components of non-exudative AMD patients with those found in a control group. Specifically, we found decreased levels of C4, IL-10, and FI in patients with non-exudative AMD. In addition, a positive correlation was observed between drusen volume and CC-chemokine ligand 4 (CCL4) levels, providing insights into potential new therapeutic targets. Finally, we investigated the relationship between biomarker levels and the demographic characteristics age, smoking status, and sex. A positive association was observed between MMP-1 and age. Additionally, smokers exhibited increased levels of CCL7 and IL-7 compared with those in non-smokers. Furthermore, significant differences in FB and C4B levels were observed between female and male probands.

## Methods

### Study design

Subjects enrolled in this case control study presented at the Department of Ophthalmology, University Medical Center Göttingen, between August 2018 and June 2021. To confirm the diagnosis of AMD or to rule out other diagnoses, all subjects underwent a detailed medical history, assessment of best-corrected visual acuity and a clinical examination of the anterior and posterior segments of the eye.

Diagnostic imaging included fundus photography, fundus autofluorescence (both Zeiss FF450 plus IR, Carl Zeiss Meditec, Jena, Germany), spectral-domain optical coherence tomography (Heidelberg Spectralis SD-OCT using 25 B-scans, Heidelberg Engineering, Heidelberg, Germany, or Zeiss Cirrus HD-5000, Carl Zeiss Meditec), and OCT angiography (OCT-A) (Zeiss Cirrus HD-5000 Angioplex or PlexElite 9000, Carl Zeiss Meditec).

Total drusen volume was determined using automated segmentation of retinal layers in the inbuilt Spectralis HRA system (Heidelberg Engineering) (Additional file 1) [38, 43]. Each scan underwent manual assessment and, if necessary, correction of the segmentation after automated segmentation. Subsequently, the drusen volume was measured between Bruch's membrane and the RPE in a 6 mm early treatment diabetic retinopathy study (ETDRS) grid. Three patients underwent OCT examinations using the Zeiss Cirrus HD-5000 device. However, drusen volume measurements were not available for these cases. As a result, total drusen volume analysis was conducted for only nine out of the twelve AMD patients.

### Population

All patients fulfilled criteria for non-exudative AMD according to Ferris et al. in the study eye [3]. They were classified as early AMD (medium-sized drusen > 63 µm and < 125 µm), intermediate AMD (large-sized drusen > 125 µm and/or retinal pigmentary changes with at least medium-sized drusen) or late AMD with geographic atrophy. No signs of AMD were present in any of the controls. All subjects were aged 50 years or older.

Subjects with choroidal neovascularisation, presence of glaucoma or any retinopathy other than non-exudative AMD in the study eye were excluded from the study. None of the subjects had a history of intravitreal injections.

The use of immunosuppressive drugs or the presence of a disease with altered systemic complement activation (e.g. systemic lupus erythematosus, malignant tumours, atypical haemolytic-uremic syndrome, membranoproliferative glomerulonephritis type II, hereditary angioedema, paroxysmal nocturnal haemoglobinuria, pyogenic infections) led to exclusion from the study.

### Collection of anterior chamber samples and protein measurement

AH was collected during routine cataract surgery. A 30-gauge insulin cannula was used to collect 150–250 µL of AH from the anterior chamber. Samples were immediately cooled to − 80 °C.

Total protein concentrations of the AH samples were determined using a bicinchoninic acid kit (Pierce, Thermo Scientific, Waltham, MA, USA). Total protein concentrations are listed in Additional file 2. No differences of protein concentrations between AMD patients and controls, females and males, or non-smokers and smokers could be observed (Additional file 3).

Parallel multiplex immunoassays based on the Luminex® xMAP® technology were conducted to detect 78 proteins in an AH sample (Table 1). For analysis and quantification of 13 complement components, the MILLIPLEX® Human Complement panels 1 and 2 (Merck, Darmstadt, Germany) were used. 12.5 µL AH were diluted in 37.5 µL assay buffer and incubated with 25 µL magnetic beads at 4 °C overnight. After three washes, 25 µL antibody detection mix was added and incubated at room temperature for 1 h, followed by 50 µL streptavidin-R-phycoerythrin for 30 min. Samples were washed thrice and measured as median fluorescence intensity (MFI) values in a Bio-Plex 200 (Bio-Rad Laboratories, Inc., Hercules, CA, USA).

**Table 1:** Determined biomarkers of this study

Group	Protein
Complement component	C1q, MBL, C2, C4, C4B, FB, FD, C3, iC3b, C5, C5a, FI, FH/FHL-1
Cytokine	G-CSF (CSF-3), GM-CSF, IFN-α, IFN-γ, IL-1α, IL-1β, IL-2, IL-3, IL-4, IL-5, IL-6, IL-7, IL-8 (CXCL8), IL-9, IL-10, IL-12p70, IL-13, IL-15, IL-16, IL-17A (CTLA-8), IL-18, IL-20, IL-21, IL-22, IL-23, IL-27, IL-31, LIF, M-CSF, MIF, TNF-α, TNF-β, TSLP
Chemokine	CCL2 (MCP-1), CCL3 (MIP-1α), CCL4 (MIP-1β), CCL7 (MCP-3), CCL8 (MCP-2), CCL11 (Eotaxin), CCL20 (MIP-3α), CCL22 (MDC), CCL24 (Eotaxin-2), CCL26 (Eotaxin-3), CXCL1 (Gro-α), CXCL5 (ENA-78), CXCL9 (MIG), CXCL10 (IP-10), CXCL11 (I-TAC), CXCL13 (BLC), CX3CL1 (Fractalkine), SDF-1α (CXCL12)
Growth factor/regulator	FGF-2, HGF, MMP-1, NGF-β, SCF, VEGF-A
Soluble factor	APRIL, BAFF, CD30, CD40L (CD154), IL-2r (CD25), TNF-RII, TRAIL (CD253), TWEAK

The Immune Monitoring 65-Plex Human ProcartaPlex™ panel (Thermo Scientific) was performed for analysis of 65 immunological factors. 12.5 µL AH were diluted in 37.5 µL assay buffer and incubated with 20 µL magnetic beads at 4 °C overnight. Two washes were followed by an incubation of 10 µL antibody detection mix with 15 µL phosphate-buffered saline for 30 min at room temperature. Samples were measured after two washes and an incubation with 50 µL streptavidin-R-phycoerythrin for 30 min.

Assay buffer was measured as a blank. Quality controls of the kits as well as a laboratory developed quality control were included in the measurements. Biomarker levels were represented in arbitrary units (AU). For the calculation of the AU, blanks were subtracted from each MFI value, divided by the mean protein concentration (mg/mL) of the respective AH sample and multiplied by 100.

### Sodiumdodecylsulfate polyacrylamide gel electrophoresis and Western blot

Western blot analyses were performed on a pooled sample of AH from AMD patients and controls, as well as on human serum, depleted human sera, and the purified proteins C4, C4b, FB, FH, FI, complement factor H-like protein 1 (FHL-1), and factor H-related protein 1 (FHR-1) (Additional file 4). Samples were prepared with reducing ROTI®Load 1 (Carl Roth, Karlsruhe, Germany) and separated by sodiumdodecylsulfate polyacrylamide gel electrophoresis (SDS-PAGE) on 4% stacking and 8% separation acrylamide gels using Bio-Rad MiniPROTEAN® (Bio-Rad Laboratories, Inc.) equipment. Proteins were transferred onto polyvinylidene fluoride membranes by wet blotting using the Mini-PROTEAN® Trans-Blot transfer system (Bio-Rad Laboratories, Inc.). After blotting, membranes were blocked for 2 h at room temperature in blocking buffer (5% milk in tris-buffered saline with Tween20). Primary antibodies were incubated in blocking buffer over night at 4 °C (Additional file 4). Secondary antibody incubation was conducted in blocking buffer at room temperature for 2 h (Additional file 4). Protein signals were detected using the WesternBright chemiluminescent substrate Sirius (Biozym Scientific GmbH, Hessisch Oldendorf, Germany) in the ChemiDoc MP Imaging System (Bio-Rad Laboratories, Inc.).

### Immune precipitation

The C4 and C4B beads of the multiplex assay kit used in this study were validated by immune precipitation with purified C4, C4b, and AH samples (Additional file 4). Kit assay buffer was used as a negative control. Each sample was incubated with 2 µL beads in assay buffer over night at 4 °C. Reducing ROTI®Load 1 was added after three washes and samples were denatured at 95 °C for 10 min. Protein mixture and beads were separated using the DynaMag™-2 Magnet (Thermo Scientific). SDS-PAGE and Western blot, as previously described Sodiumdodecylsulfate polyacrylamide gel electrophoresis and Western blot, were performed.

### Graphical and statistical analysis

Graphical and statistical analysis was conducted using R version 4.2.1 [44] and the R packages tidyverse [45], pastecs [46], car [47], ggsignif [48], RColorBrewer [49], gmodels [50], and polycor [51]. Normality was assessed with histograms, Q-Q plots, values for kurtosis and skew, as well as Shapiro Wilk’s test. Homogeneity of variance was checked with Levene’s test.

Heatmaps of each protein were created to depict general biomarker levels, represented as adapted arbitrary unit (AAU), using GraphPad Prism Version 9.3.1 (GraphPad Software, Inc., La Jolla, CA, USA). For the calculation of the AAU and adjustment to the detection limits, the mean AU for parametric datasets and the median AU for non-parametric datasets was multiplied by the minimum detection level (pg/mL) of each protein, stated in the certificate of analysis of the respective kit lot numbers (Additional file 5). Proteins were separated according to the magnitude of biomarker levels. An AAU ≥ 10 * 10^5^ was designated as a high level, 10 * 10^5^ > AAU ≥ 1 * 10^3^ as a medium level, and AAU < 1 * 10^3^ as a low level.

Pearson’s chi-square test was used to analyse if sex or smoking status significantly influenced if a subject had AMD or not. Independent-means *t*-test was used to compare age between control vs. patient group. Biomarker levels were compared between the groups control vs. patient, smoker vs. non-smoker, and male vs. female. Levels of CCL7, IL-7, and IL-6 were compared between smokers with and without AMD in a subgroup analysis. For parametric data, an independent-means *t*-test was conducted and mean (M) as well as standard deviation (SD) are depicted in the graphs and tables. For non-parametric data, Wilcoxon’s rank-sum test was used, while median (Mdn) with first (Q_1_) and third (Q_3_) quartile are depicted in graphs and tables. Tests were two-tailed and an alpha level of 0.050 (*P* < 0.050) defined statistical significance. Furthermore, effect size *r* is listed in the tables (Additional files 7–11). In the group of AMD patients, a Spearman’s correlation with the correlation coefficient *r*_*S*_ was used to correlate drusen volume with biomarker levels. Spearman’s Correlation was further used to correlate the biomarker levels with subject age. An alpha level of 0.050 indicated significance of *r*_*S*_.

To assess whether age introduced bias when comparing biomarker levels of IL-10, C4, and FI between AMD patients and controls, we performed regression analysis (Additional file 6). First, a simple regression analysis was conducted with AMD status as the predictor for the biomarker level, evaluating effect size (*R*^*2*^), F-statistic, and *P* value. Subsequently, age was added as a second predictor in a multiple regression analysis to determine if this improved the model. The same approach was applied to investigate whether AMD status biased the correlation of age with biomarker levels of MMP-1 and CCL20. As no significant model improvement was observed in either case, we concluded that age and AMD status did not influence the respective analyses.

## Results

### Detected proteins in the AH

**Fig. 1:**
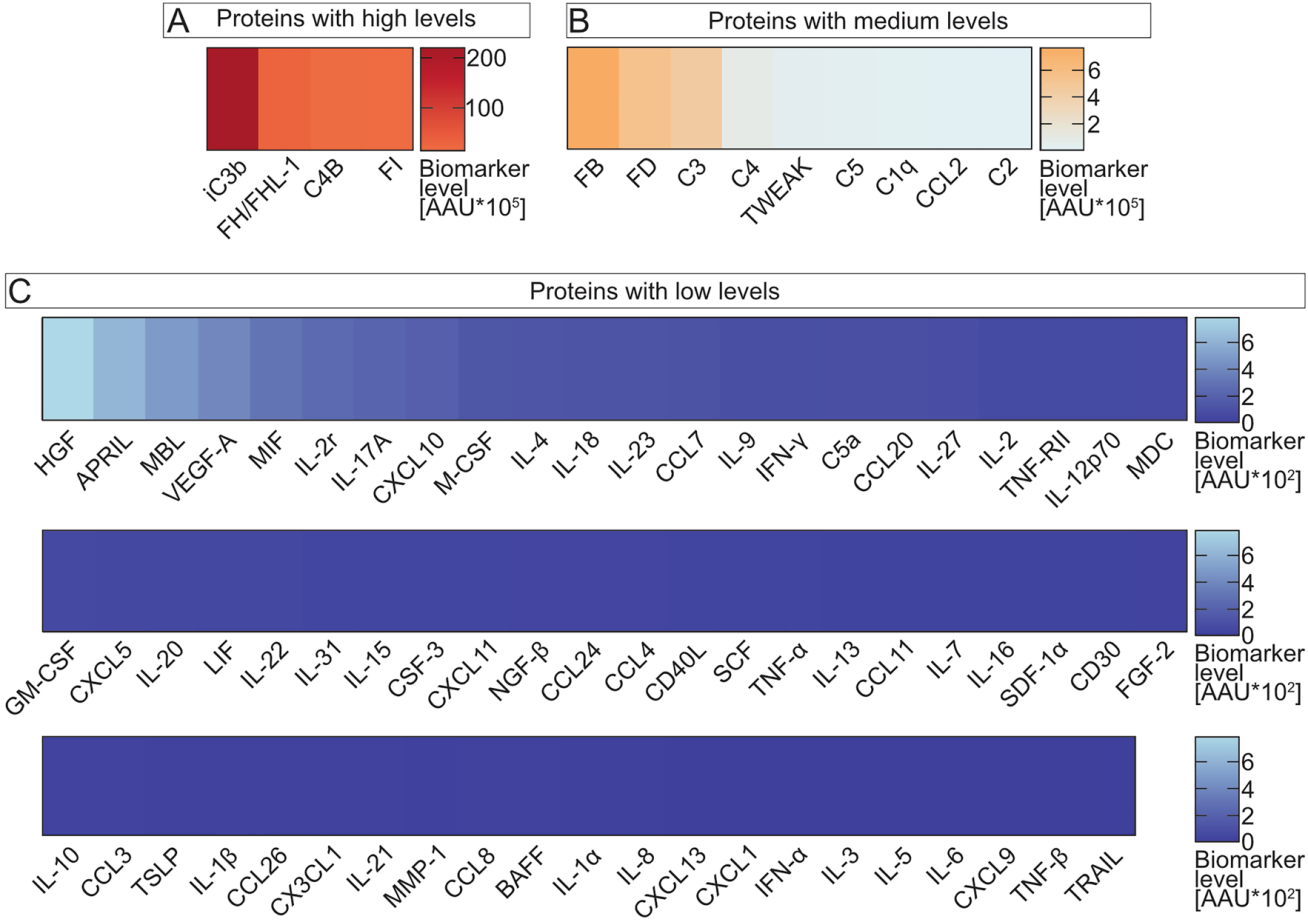
Categorisation of 78 proteins in the AH of all subjects. Heatmaps represent the average biomarker levels in all subjects, adjusted to the kit detection limits, in descending order of adapted arbitrary unit (AAU) levels (data in Additional file 5). **A** iC3b, FH/FHL-1, C4B, and FI displayed the highest AH levels (AAU ≥ 10 * 10^5^), **B** FB, FD, C3, C4, TWEAK, C5, C1q, CCL2, and C2 showed medium levels (10*10^5^ > AAU ≥ 1 * 10^3^) and **C** 63 proteins were detected with low levels (AAU < 1 * 10^3^). Protein measurements were conducted using Luminex® xMAP® technology. *n* = 33 subjects

The study cohort comprised a total of 33 subjects (12 non-exudative AMD patients and 21 healthy controls). Their demographic characteristics are depicted in Table 2. Early AMD status was seen in five patients, intermediate in six patients and geographic atrophy in one patient. Biomarker levels of 78 immunological factors were quantified in AH of both the patient and control groups (Fig. 1A–C). In summary, 4 proteins exhibited a high level (complement iC3b (iC3b), FH/FHL-1, C4B, FI), while 9 proteins displayed a medium level (FB, complement factor D (FD), C3, C4, tumour necrosis factor-like weak inducer of apoptosis (TWEAK), complement 5 (C5), complement 1q (C1q), CC-chemokine ligand 2 (CCL2), complement 2 (C2)). Furthermore, 65 proteins demonstrated a comparatively low biomarker concentration.

**Table 2:** Summary of subject demographics

Number	AMD patients	Controls	*P*
	12	21	
Female/male	5/7	8/13	0.840
Age (years (SD))	80.17 (6.00)	71.71 (8.15)	0.002*
Female	80.20 (5.04)	75.38 (6.82)	
Male	80.14 (6.20)	69.46 (7.76)	
Non-smoker/smoker^†^	7/4	13/7	0.939
Female	3/1	6/1	
Male	4/3	7/6	

*P* value refers to the comparison of AMD status by independent-means *t*-test of age or Pearson’s chi-square test between males and females, or non-smokers and smokers. Mean (M) with standard deviation (SD) or number are depicted, * *P* < .050 represents significance, ^†^ Smoking status was not available of two subjects

### Comparative analysis in AMD patients and controls revealed distinct C4, IL-10, and FI

**Fig. 2:**
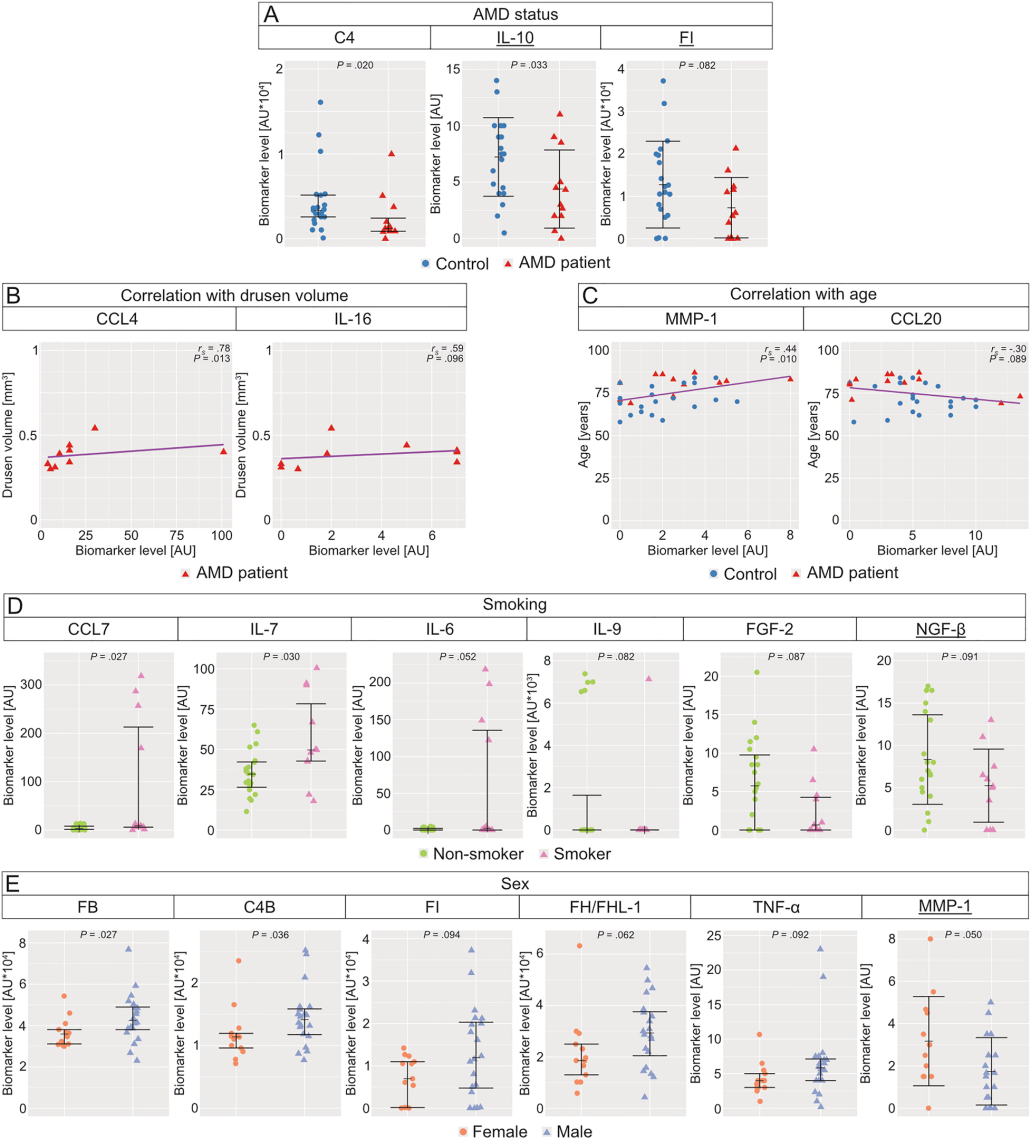
AH protein profiling in relation to AMD status, drusen volume, age, smoking, and sex. Biomarker level differences in the AH were assessed. **A** Differences were observed for C4, IL-10, and FI between AMD patients (*n* = 12) and controls (*n* = 21). **B** Correlations of the levels of CCL4 and IL-16 with drusen volume in AMD patients (*n* = 9) are displayed. **C** Correlations of MMP-1 and CCL20 with subject age (*n* = 33) were observed. Datapoints for AMD patients and controls are indicated in their respective colours. **D** Highlighted are biomarker level differences of CCL7, IL-7, IL-6, IL-9, FGF-2, and NGF-β between smokers (*n* = 11) and non-smokers (*n* = 20). **E** Differences of biomarker levels were identified for FB, C4B, FH/FHL-1, TNF-α, FI, and MMP-1 between females (*n* = 13) and males (*n* = 20). Data for underlined proteins underwent parametric analysis (independent-means *t*-test, mean (M) and standard deviation (SD) are displayed), whereas the rest underwent non-parametric analysis (Wilcoxon’s rank-sum test, median (Mdn) with interquartile range are displayed). Protein measurements were performed using Luminex® xMAP®

A comparison of biomarker levels was conducted between AMD patient and control cohorts (Fig. 2A, Additional file 7). A difference with a *P* value below 0.100 was observed for three proteins. Specifically, C4 (*P* = 0.020) and IL-10 (*P* = 0.033) exhibited a statistically significant reduction in the AMD group relative to the control group. While FI (*P* = 0.082) showed a decrease in the AMD group compared with the control group, this difference did not achieve statistical significance. The remaining biomarker levels showed no differences between the AMD and control groups (Additional file 7). To rule out the potential influence of age as a confounding factor in the analysis, we performed statistical testing and found no evidence of age-related bias in the observed results (Additional file 6).

### Correlation between drusen volume and biomarker levels identified CCL4 and IL-16 as positively associated

As a hallmark of AMD, the presence of drusen was only detected in the AMD group, with a mean total drusen volume of 0.38 ± 0.03 mm^3^. However, it should be noted that the drusen volume was only determined in nine out of twelve AMD patients. Correlations between all biomarkers and drusen volume were examined, with key biomarker correlations shown in Fig. 2B. The biomarker level of CCL4 (*r*_*S*_ = 0.78, *P* = 0.013) demonstrated a significant positive correlation with the drusen volume. Moreover, IL-16 (*r*_*S*_ = 0.59, *P* = 0.096) exhibited a positive correlation with the drusen volume, although this correlation did not reach statistical significance. Conversely, the remaining biomarker levels exhibited no notable correlations with the drusen volume (Additional file 8).

### MMP-1 and CCL20 levels exhibited correlations with proband age

The analysis of biomarker levels regarding the subjects’ age is shown in Fig. 2C. A significant positive correlation was identified between age and MMP-1 (*r*_*S*_ = 0.44, *P* = 0.010). Additionally, a negative correlation was observed for CCL20 (*r*_*S*_ = -0.30, *P* = 0.089), although this correlation did not reach statistical significance. The other biomarker levels did not exhibit any noteworthy correlations with age (Additional file 9). To rule out the potential influence of AMD status as a confounding factor in the analysis, we performed statistical testing and found no evidence of disease-related bias in the observed results (Additional file 6).

### Distinct concentrations of the six biomarkers CCL7, IL-7, IL-6, IL-9, FGF-2, and NGF-β between smokers and non-smokers detected

Regarding the smoking status, individuals who smoked demonstrated a significant increase in the biomarker levels of CCL7 (*P* = 0.027) and IL-7 (*P* = 0.030) in comparison with those in non-smokers (Fig. 2D, Additional file 10). Moreover, four additional proteins exhibited differences with a *P* value below 0.100. Specifically, levels of IL-6 (*P* = 0.052) were increased, while levels of interleukin-9 (IL-9) (*P* = 0.082), FGF-2 (*P* = 0.087), and nerve growth factor beta (NGF-β) (*P* = 0.091) were decreased in smokers compared with those in non-smokers. However, no differences were observed for the remaining proteins (Additional file 10). Furthermore, subgroup analysis comparing smokers with and without AMD showed no significant differences in the levels of CCL7, IL-7, and IL-6 (Additional file 10).

### Sex-based comparison of biomarker levels revealed the six notable proteins FB, C4B, MMP-1, FH/FHL-1, TNF-α, and FI

Figure 2E presents the analysis of biomarker levels in relation to the sex of the subject (Additional file 11). FB (*P* = 0.027) and C4B (*P* = 0.036) demonstrated significant lower levels in females as compared with those in males. Additionally, FH/FHL-1 (*P* = 0.062), TNF-α (*P* = 0.092), and FI (*P* = 0.094) exhibited a decrease, while MMP-1 (*P* = 0.050) showed an increase in females compared with males. However, these differences did not reach statistical significance. Among the six relevant proteins, four (C4B, FB, FH/FHL-1, and FI) belonged to the group of complement components. No further differences were observed for the remaining proteins (Additional file 11).

### Validated presence of complement components in the AH

The presence of distinct complement components in the AH was further validated (Additional file 12). This included C4, C4B, FB, FI, and FH, each linked to significant differences with a *P* value below 0.100 either in relation to AMD status or sex comparisons conducted within this study (Fig. 2A, E). This analysis was conducted on an AH pool comprising samples from both AMD patients and controls. All target components could be identified in the AH, human serum, and purified proteins (Additional file 12A, B, E–I). In contrast, depleted sera exhibited no signals, confirming the specificity of the detection methods. Immune precipitation of the multiplex assay kit C4B beads showed a higher sensitivity in comparison with C4 bead-precipitation, since the C4 α- and β-chain were only detectable in C4B bead-precipitated AH (Additional file 12C, D).

Validating C4-specific beads using purified antigens showed bead types in Panels 1 and 2 detect both inactive C4 and active C4b. Tests with recombinant C4 fragments (including aa 1120–1125) revealed Panel 2 beads favor the C4B isoform, while Panel 1 beads aren’t isoformspecific (Additional file 12J,K). Furthermore, validation of the multiplex immunoassays demonstrated that the FH beads included in the multiplex assay kit couldn’t differentiate between FH and FHL-1 (Additional file 12N). Therefore, the higher levels of FH and FHL-1 in males compared with those in females could be attributed to both proteins and differentiation between those proteins was not possible.

## Discussion

### Case control study for a comprehensive biomarker analysis in AH

Given the existing demand for treatment options in non-exudative AMD, the exploration of novel therapeutic target proteins is of great interest. A pilot study was conducted to comprehensively determine inflammatory factors related to the non-exudative form of AMD and identify potential targets for future biomarker studies. This study investigated the presence of 78 immunological factors in the AH of non-exudative AMD patients and controls, providing valuable insights. The analysis was conducted using the Luminex xMAP technology. In addition, complement proteins of interest were further validated through Western blot experiments to verify their appearance in the AH. It is important to recognise the small sample size, consisting of 12 non-exudative AMD patients and 21 controls, positioning it as an initial pilot investigation. A second limitation was the significant age difference between the AMD and control cohorts. However, our analyses demonstrated that this age disparity did not introduce bias in the conducted statistical evaluations (Additional file 6). Despite this limitation, our study demonstrated significant differences and correlations as well as trends within our study groups.

It is worth noting that all 78 examined inflammatory mediators and complement components were detectable in the AH of the subjects. Among several proteins within the subgroup of low levels, certain probands displayed MFI values of zero, indicating levels below the detection limit. However, as the rest of the probands exhibited values above the detection limit, these specific proteins were not excluded, but rather included in the low biomarker level group (Additional file 5). Nevertheless, it is important to highlight that alternative methods should distinctly validate their presence within the AH.

### Influence of inflammatory processes in the retina on AH in the anterior chamber

Based on the abundant presence of various inflammatory mediators within the AH, it becomes evident that the anterior chamber of the eye is susceptible to multiple different inflammatory processes. The question arises as to whether inflammation, which is associated with a disease such as AMD, that occurs in the posterior segment of the eye, can affect the AH in the anterior chamber. Recent studies have assessed this issue and conducted analyses of proteins of AH from the anterior chamber and vitreous humour (VH). Studies focusing on proliferative diabetic retinopathy observed notable associations of the two complement activation products complement 3a (C3a) and complement factor Ba (Ba) as well as VEGF and IL-6 within both ocular fluids [52, 53]. Their findings align with the outcome of Noma et al., who investigated VEGF and IL-6 in the context of branch retinal vein occlusion and reported similar significant correlations [54]. Furthermore, Wilson et al. identified strong correlations within the AH and VH among 490 proteins out of over 800 proteins analysed [55]. These findings suggest that protein alterations associated with retinal diseases can indeed be observed in the AH.

### Challenges when comparing biomarker results due to low number of AH studies in non-exudative AMD and differences in study designs

Several studies with a similar objective to compare inflammatory proteins in AH from AMD patients and controls have previously been conducted. While few studies have specifically examined the AH of non-exudative AMD patients and controls [38–42], the majority have focused on exudative AMD [56–73]. The evident discrepancy in the quantity of AH biomarker studies between exudative and non-exudative AMD highlights the need for more analyses in the latter form. Furthermore, previous studies using immunoassay-based methods primarily focused on a smaller subset of proteins. Therefore, our study distinguishes itself by the analysis of a comprehensive array of 78 distinct inflammatory factors. It is important to acknowledge that the comparison of our study’s findings with those of prior investigations is challenged by variations in experimental designs, analytical methodologies, and diagnostic techniques. Notably, our study employed OCT-A as a diagnostic modality, which is renowned for its ability to detect early microvascular impairment [74]. With this, we aimed to reduce the risk of misclassifying subclinical macular neovascularisation as non-exudative. OCT-A was shown to have good sensitivity and specificity for detecting choroidal neovascularisation compared with the more invasive fluorescein angiography [75].

### C4 with diverse associations to AMD

While the role of inflammation and the complement system in the development and progression of AMD is established, its detailed pathomechanisms still remain to be elucidated. Within our study, we identified three proteins that were differently concentrated in the AH of non-exudative AMD patients and controls. Interestingly, the two complement components C4 and FI as well as IL-10 exhibited lower levels in the AH of AMD patients compared with those in controls.

C4 serves as a central protein in initiating both the classical and lectin pathways of the complement system. Its activation by complement 1r (C1r) and complement 1s (C1s) or by mannan-binding lectin-associated serine proteases (MASPs) generates the cleavage products C4a and C4b. C4b contributes to the assembly of one of the C3 convertases, subsequently promoting C3 activation. Finally, the terminal pathway is initiated, leading to the formation of the membrane attack complex [15, 16]. C4 is encoded by two gene isoforms, complement 4A (*C4A*) and 4B (*C4B*). The translated proteins, C4A and C4B, differ only by four amino acids, localised in the complement 4d (C4d) fragment of the α-chain [76, 77]. Despite this small difference, C4A and C4B demonstrate distinct functionalities. C4A displays a higher affinity for binding free amino groups, whereas C4B exhibits a preference for hydroxyl groups [78, 79].

In our study, we observed higher detectable levels of soluble C4 in the AH of controls compared with those in AMD patients. However, no significant difference was observed in the total C4B level between controls and AMD patients. While it may be hypothesised that AMD patients display decreased C4A levels in the AH, experimental validation of this theory has yet to be conducted. In previous studies, C4B has not been specifically analysed in the AH of non-exudative AMD patients. However, a study of Sitnilska et al. measured C4B in the AH of early AMD patients and controls with the same multiplex immunoassay used in our study. Consistent with our study, no differences in C4B concentrations were observed [38]. Furthermore, Zauhar et al. reported an increase *C4B* gene expression in the macular and a decrease in the peripheral retina of late-stage AMD patients compared with healthy subjects and early AMD forms [80].

Genetic association studies have revealed that rare polymorphisms within *C4A* exert both protective and risk-increasing effects for AMD [19]. Increased copy numbers of *C4A* have previously been associated to a protective effect for the development of late-stage AMD [81]. The extent to which genetic findings can be linked to protein quantity and function, particularly for this highly polymorphic complement protein C4, needs to be elucidated in future investigations. Nevertheless, studies have demonstrated that C4A and C4B are deposited in tissue by distinct mechanisms, thus competitively influencing the degradation of synapses in the brain [82, 83]. It is important to note that our analysis only considers the soluble concentration in AH and does not include interactions with tissue. Therefore, the observed lower C4 concentrations may indicate heightened C4 deposition in the tissue of AMD patients compared with other diseases. This underscores the importance of additional research to fully understand the relationship between C4 levels and AMD.

### FI as a regulator to dampen complement activation during AMD progression

As mentioned above, we detected decreased levels of FI in AMD patients. Two comparable studies conducted by Sitnilska et al. and Hallam et al. analysed the AH of patients with early or non-exudative AMD, respectively. Overall, neither study reported significant differences in FI levels between patient and control groups [27, 38]. However, Hallam et al. indicated the trend of a reduced FI concentration in the AH of the non-exudative AMD group compared with controls, which aligns to our findings, though their study was also limited by a small sample size for AH analysis [27].

FI is a central regulator of the complement system, among other functions it is involved in degenerating the activation products C3b and C4b [17]. Numerous genetic variants of *CFI* have been described that significantly increase the risk of developing advanced AMD. Many of those variants result in reduced FI secretion in serum or plasma and consequently increase complement activation [20–24, 26, 27]. Our findings align with the hypothesis that diminished FI presence and function in our AMD patients could contribute to elevated complement system activation, thereby potentially increasing the susceptibility to develop non-exudative AMD. Further investigations of functional genetic variants within *CFI* could provide insights into the cause of reduced FI levels in our AMD patients. The prospect of targeting FI to potentially dampen complement activation may offer a viable approach for the treatment of AMD. However, clinical trials of a genetic FI replacement therapy in geographic atrophy patients have been discontinued (NCT03846193, NCT04437368, NCT04566445).

### The role of IL-10 to possibly increase AMD progression

Furthermore, we observed decreased levels of IL-10 in the AH of non-exudative AMD patients in comparison with those in controls. A study of Spindler et al. examined IL-10 levels in the AH of non-exudative AMD patients and controls. Their findings revealed a contradictory trend, with increased IL-10 levels observed in their non-exudative AMD cohort, although this difference was not significant [39]. Furthermore, Kramer et al. and ten Berge et al. reported no differences in IL-10 in the AH of AMD patients [40, 41]. However, it is noteworthy that their AMD patient groups included exudative AMD patients, diverging from our specific focus on non-exudative AMD patients.

In general, IL-10 is known to be a cytokine with anti-inflammatory and immunosuppressive functions [84]. Therefore, the reduced anti-inflammatory functionality of IL-10 observed in our AMD patients could potentially contribute to increased inflammation and subsequent progression of AMD. Targeting an anti-inflammatory cytokine locally in the retina could hold promise as a potential therapeutic strategy for AMD to suppress further inflammatory processes.

### Association between drusen volume and CCL4 as well as IL-16 levels in relation to AMD progression

We observed a positive correlation between the AH concentration level of the chemokine CCL4 and the cytokine IL-16 with the volume of retinal drusen. Several research groups have already conducted proteomic analyses of drusen in AMD, revealing the presence of proteins of various pathways. Notably, proteins associated with the complement pathway, including FH or C9, have been identified among the constituents of drusen [31–34]. However, no study specifically described CCL4 or IL-16 in drusen or in relation to drusen volume yet.

Increased drusen volume indicates advanced progression of AMD and associated inflammatory processes, potentially linked to the elevated biomarker levels of CCL4 and IL-16 we observed. A study of Joo et al. demonstrated significantly increased CCL4 levels in the AH of exudative AMD patients compared with those in controls [72]. Similar to non-exudative AMD, exudative AMD is characterised by the development and growth of drusen, which aligns with our findings. Furthermore, our observation strengthens the hypothesis that pathological processes occurring within the retina can indeed influence the concentration of inflammatory mediators in the AH.

### Differences regarding demographic characteristics

As a secondary objective, we analysed differences in biomarker levels concerning demographic characteristics such as age, smoking status and sex. Due to the limited sample sizes within the distinct cohorts, this analysis was performed across the entire study population without stratifying by AMD status. Consequently, results were interpreted without taking into consideration whether the proband had AMD or not.

#### Age-related pathological processes linked to increased function of MMP-1

In the study conducted by Cai et al., an analysis of gene expression in both the macular and peripheral retina across different age groups unveiled numerous age-dependent transcriptomic variances [85]. Our study specifically identified a positive correlation between the regulator MMP-1 and age, consistent with a prior study examining the AH of diabetic macular oedema patients and controls [86]. Additionally, we observed a negative correlation between the chemokine CCL20 and age, which has not been previously analysed in existing studies.

Given the significant influence of age as a major demographic risk factor for AMD, age-dependent alterations in protein secretion may serve as initiating factors in AMD development [87]. Proteinase MMP-1 is involved in the breakdown of all components of the extracellular matrix [88]. Under normal conditions, healthy tissues exhibit low levels of MMP-1, but inflammation, as it takes place during ageing, leads to an increase in its secretion. This has for example been demonstrated by the upregulation of MMP-1 in oxidatively stressed RPE cells [10]. Extending beyond the retinal location, a study of Fisher et al. observed increased levels of MMP-1 in dermal skin fibroblasts of aged compared with young probands. Additionally, treatment with MMP-1 induced structural changes in skin fibroblasts that were similar to those observed during ageing [89]. Consistent with these findings, our study revealed a positive correlation between elevated MMP-1 levels and older age among the participants. This observation supports the hypothesis that during the ageing process and structural inflammatory alterations in the retina, increased secretion of MMP-1 could be induced, thereby contributing to the development and progression of AMD. Additionally, the increased MMP-1 secretion is detectable in the AH.

#### Smoking-associated inflammation is locally detectable in the AH

A notable discovery emerges from the comparison of smokers with non-smokers, with the latter group including both non-smokers and former smokers. In smokers, we detected elevated levels of CCL7, IL-7, and IL-6 in comparison with those in non-smokers. It’s well established that smoking is linked to a systemic inflammatory response [90]. However, the impact of smoking on AH has been less explored. A comprehensive proteomic analysis conducted by Amer et al. identified 67 proteins that were either up- or down-regulated in the AH of chronic smokers compared with non-smokers [91]. This included several complement components, such as C4A, C4B, FB, and complement factor H-related protein 3 (FHR-3). However, they did not observe differences in the specific proteins that our study identified. Furthermore, previous studies correlated increased IL-6 in the AH of patients with diabetic retinopathy or primary open-angle glaucoma to smoking [11, 92]. In general, our results support these findings by revealing the upregulation of three inflammatory proteins in the AH of smokers. This suggests that inflammation associated with smoking is locally detectable in the AH.

#### Differences in complement activity between females and males

We further conducted a comparative analysis of protein concentrations between females and males. Among the six proteins of interest, four belonged to the complement pathway, specifically C4B and the complement regulators FB, FH/FHL-1, and FI. Notably, these components exhibited decreased levels in females compared with those in males. Although no previous studies have directly compared these proteins in the AH of females and males, Marin et al. investigated this aspect in the plasma of intermediate AMD patients or controls. However, their observations do not align with the results observed in our study [93]. But in contrast to our study, Marin et al. conducted separate comparisons between females and males within their intermediate AMD and control cohorts, exacerbating the direct comparison. Another investigation by Gaya de Costa et al. observed trends in serum, indicating lower activity in the alternative and classical complement pathways among females compared with males [94]. However, they did not observe differences in C4 or FB, while FH and FI were not specifically analysed.

In addition to the complement components, our study revealed an increase in MMP-1 and a decrease in the cytokine TNF-α among females. It is noteworthy that no studies have been conducted to date comparing sex differences in the AH for these specific proteins. Although one study investigated MMP-1 in plasma in the context of infectious conditions, it did not identify differences between females and males [95].

## Conclusion

In conclusion, we measured 78 proteins in AH and further identified proteomic associations with non-exudative AMD and its clinical characteristics. Specifically, decreasing levels of complement components C4 and FI as well as the cytokine IL-10 were observed within the AH of AMD patients. Correlations between biomarker levels, drusen volume, and demographic characteristics were established, including age, smoking status, and sex. Drusen volume positively correlated with CCL4 levels, while MMP-1 was positively associated with age. According to expectation, smoking resulted in an elevation in the levels of pro-inflammatory cytokines, specifically CCL7 and IL-7. Finally, differences regarding sex were observed for FB and C4B. In summary, we could locally detect disease-associated changes of inflammatory mediators and complement components in the AH. This study served as a pilot investigation, providing an initial overview of biomarkers in the AH of non-exudative AMD patients. A more focused follow-up study with a larger sample size and stratified cohorts is currently underway to further investigate these targeted biomarkers. Our findings underline the important role of inflammatory processes and dysregulation of the complement system in the pathogenesis and progression of non-exudative AMD.

## Supplementary Information

Additional file 1. Semi-automated drusen volume measurement using the Spectralis HRA system by Heidelberg engineering. **(A)** Infrared fundus image with heat map of drusen volumes in the 6 mm ETDRS grid. **(B) **Mean drusen thickness (black numbers, µm) and corresponding drusen volume (red numbers, mm^3^) of each ETDRS subgrid. **(C)** Drusen volume is measured in each OCT scan between Bruch's membrane (BM) (red line) and the retinal pigment epithelium (RPE) (blue line). **(A)**, **(B)**, and **(C)** show an exemplary evaluation for a non-exudative AMD patient.

Additional file 2. Total protein concentrations in AH of subjects. Total protein concentrations (mg/mL) of the AH samples were determined using a bicinchoninic acid protein assay.

Additional file 3. Comparison of protein concentrations among various cohorts. It was determined if notable differences existed in protein concentrations across the diverse groups of patients (n = 12) and controls (n = 21), females (n = 13) and males (n = 20), as well as non-smokers (n = 20) and smokers (n = 11). No statistically significant differences were observed among these groups. This outcome validates the suitability of using protein concentrations for the normalisation of data obtained from the multiplex immunoassay. A comparison of AMD status and sex by independent-means t-test was conducted, while Mann-Whitney test was used for comparison of smoking status. For AMD status and sex, mean (M) and standard deviation (SD) are depicted, while median (Mdn) and first (Q_1_) and third quartile (Q_3_) are depicted for smoking status. ^†^ Smoking status was not available of two subjects.

Additional file 4. Antibodies and proteins used in validation experiments (Additional file 12). Listed are concentrations/dilutions and references of the used antibodies, purified proteins, sera and the amount of AH used in validation experiments. Primary antibodies anti-FH.15 and anti-FH.16 were provided by Sanquin Research (Amsterdam, Netherlands) [96]. Recombinant FHL-1 402Y and 402H variants were produced by Józsi [97].

Additional file 5. Original data of average biomarker levels underlying heatmap presentation. Displayed are average biomarker levels in all subjects, that were adapted to the kit detection limits, in descending order of adapted arbitrary unit (AAU). For the calculation of the AAU, the mean arbitrary unit (AU) for parametric datasets or the median AU for non-parametric datasets was multiplied by the minimum detection level (pg/mL) of each protein, that was given by the certificate of analysis of each kit. Underlined proteins were parametric. Proteins were separated according to the magnitude of AAU levels. An AAU ≥ 10*10^5^ was labelled as a high level, 10*10^5^ > AAU ≥ 1*10^3^ as a medium level and AAU < 1*10^3^ as a low level. Some proteins included in the low level group showed probands with MFI values of zero below the detection limit. Since most probands exhibited values that exceeded the detection limit, these specific proteins were not excluded, but included in the low biomarker level group.

Additional file 6. Assessment of potential bias from age and AMD status in biomarker analyses. Simple regression analysis was performed with AMD status as the predictor for biomarker levels of IL-10, C4, and FI, evaluating *R²*, F-statistic, and *P* values. Age was then added as a second predictor in a multiple regression to assess model improvement. The same analysis was conducted to evaluate the potential bias of AMD status on the correlation between age and biomarker levels of MMP-1 and CCL20. No significant improvement was observed in either case, indicating that age and AMD status did not influence the respective analyses. * *P* < .050 represents significance.

Additional file 7. Summary of biomarker levels of patients and controls. A comparison between biomarker levels of AMD patients (n = 12) and controls (n = 21) was conducted. Parametric data underwent independent-means t-test, presented as arbitrary unit (AU) with mean (M) and standard deviation (SD). Non-parametric data was analysed by Wilcoxon’s rank-sum test, depicted as AU with median (Mdn) with first (Q_1_) and third (Q_3_) quartile. * P < .050 represents significance.

Additional file 8. Correlation of drusen volume and biomarker levels. Drusen volume and biomarker levels were correlated in the patient group using Spearman’s correlation with the correlation coefficient r_S_. * P < .050 represents significance. n = 9.

Additional file 9. Correlation of age and biomarker levels. Age and biomarker levels were correlated in the whole proband group using Spearman’s correlation with the correlation coefficient r_S_. * P < .050 represents significance. n = 33.

Additional file 10. Summary of biomarker levels of smokers and non-smokers. A comparison between biomarker levels of smokers (n = 11) and non-smokers, including former smokers, (n = 20) was conducted. The second tab shows the subgroup analysis of CCL7, IL-7, and IL-6 between smokers with and without AMD. Parametric data underwent independent-means t-test, presented as arbitrary unit (AU) with mean (M) and standard deviation (SD). Non-parametric data was analysed by Wilcoxon’s rank-sum test, depicted as AU with median (Mdn) with first (Q_1_) and third (Q_3_) quartile. * P < .050 represents significance.

Additional file 11. Summary of biomarker levels of females and males. A comparison between biomarker levels of females (n = 13) and males (n = 20) was conducted. Parametric data underwent independent-means t-test, presented as arbitrary unit (AU) with mean (M) and standard deviation (SD). Non-parametric data was analysed by Wilcoxon’s rank-sum test, depicted as AU with median (Mdn) with first (Q_1_) and third (Q_3_) quartile. * P < .050 represents significance.

Additional file 12. Complement protein detection by Western blot and relevant specificity controls for multiplex immunoassay. (**A**, **B**) The C4 α-chain and activated C4b α’-chain were identified in purified proteins, serum and AH. (**B**) Assessment of C4 subdomains (α-, β-, γ-chains) using polyclonal antiserum detected specific bands in purified proteins, serum, and AH. (**C**, **D**) Immune precipitation with C4B beads, but not C4 beads revealed the C4 α- and β-chain in AH. No signals were detectable in the negative control (neg ctrl). (**E**) Specific bands for FB were observable in purified FB, human serum, and AH. (**F**) Presence of FI was confirmed in purified FI, human serum, and AH, while it was absent in FI-depleted serum. (**G**) A specific antibody targeting complement control protein 19 of FH identified FH in a positive control, human serum, and AH. No signals were observed in FH-depleted serum or in purified FHL-1, FH-related (FHR)-1, and FHR-3 proteins. (**H**) Detection of FH and FH-associated proteins was performed with an antibody targeting complement control protein 5. It was exclusively found in purified FH and FHL-1, while no signals were observed for FHR-1 and FHR-3. (**I**) Analysis with polyclonal antiserum revealed a band for FH, FHL-1, and a distinct double band, consistent with reported signals for FHR-1. A band for FH was observable in AH. (**J**–**N**) Specificity controls were performed using the Luminex® xMAP® technology. (**J**, **K**) C4 beads as well as C4B beads identified purified C4, C4b, and human serum, while lower signals were observed in C4-depleted serum. Recombinant C4B was detect by C4-specific beads from panel 2. (**L**) FB was detected in purified FB and serum with FB-specific beads. (**M**) FI beads showed signals in purified FI and serum samples, but no signal in FI-depleted serum. (**N**) FH beads detected FH and both FHL-1 402Y and 402H variants, while no signals were observed for other FH-related components and FH-depleted serum. sec = exposure time in seconds.

## Data Availability

All data generated or examined during this research study are included within this published article and its supplementary materials. Additional information can be obtained upon reasonable request from the corresponding author.

## Abbreviations

AAU: Adapted arbitrary unit
AMD: Age-related macular degeneration
AU: Arbitrary unit
AH: Aqueous humour
CCL2: CC-chemokine ligand 2
CCL4: CC-chemokine ligand 4
CCL7: CC-chemokine ligand 7
CCL20: CC-chemokine ligand 20
C1q: Complement 1q
C1r: Complement 1r
C1s: Complement 1s
C2: Complement 2
C3: Complement 3
C3a: Complement 3a
C3b: Complement 3b
C4: Complement 4
C4a: Complement 4a
C4A: Complement 4A
C4b: Complement 4b
C4B: Complement 4B
C4d: Complement 4d
C5: Complement 5
C9: Complement 9
FB: Complement factor B
Ba: Complement factor Ba
FD: Complement factor D
CFH, FH: Complement factor H
FHL-1: Complement factor H-like protein 1
FHR-1: Complement factor H-related protein 1
FHR-3: Complement factor H-related protein 3
CFI, FI: Complement factor I
iC3b: Complement iC3b
ETDRS: Early treatment diabetic retinopathy study
*r*: Effect size
FGF-2: Fibroblast growth factor 2
Q_1_: First quartile
IL-6: Interleukin-6
IL-7: Interleukin-7
IL-9: Interleukin-9
IL-10: Interleukin-10
IL-16: Interleukin-16
IL-18: Interleukin-18
MASP: Mannose-binding lectin (MBL)-associated serine protease
MMP-1: Matrix metalloproteinase-1
M: Mean
Mdn: Median
MFI: Median fluorescence intensity
NGF-β: Nerve growth factor beta
OCT: Optical coherence tomography
RPE: Retinal pigment epithelium
SDS-PAGE: Sodiumdodecylsulfate polyacrylamide gel electrophoresis
*r*_*S*_: Spearman’s correlation coefficient
SD: Standard deviation
Q_3_: Third quartile
TNF-α: Tumour necrosis factor alpha
TWEAK: Tumour necrosis factor-like weak inducer of apoptosis
VEGF: Vascular endothelial growth factor
VH: Vitreous humour
