# Supplementary figures and images for: Decreased complement 4 and interleukin-10 as biomarkers in aqueous humour for non-exudative age-related macular degeneration: a case control study
Source: J Transl Med. 2025 Mar 12;23:317. doi: 10.1186/s12967-024-05909-x (PMC11905602; doi:10.1186/s12967-024-05909-x)

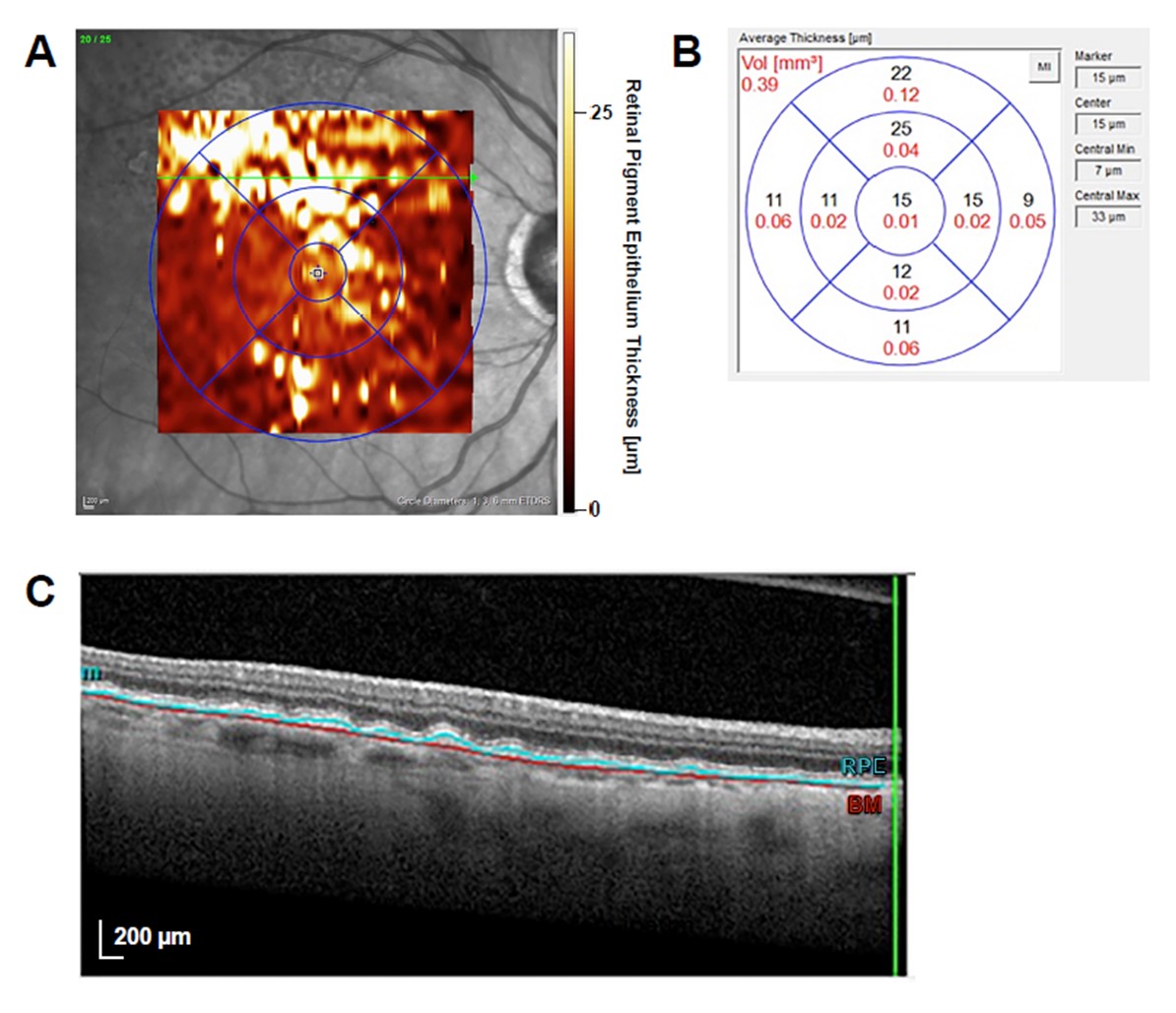

Supplement: Supplementary file 1 — Additional file 1. Semi-automated drusen volume measurement using the Spectralis HRA system by Heidelberg engineering. (A) Infrared fundus image with heat map of drusen volumes in the 6 mm ETDRS grid. (B) Mean drusen thickness (black numbers, µm) and corresponding drusen volume (red numbers, mm3) of each ETDRS subgrid. (C) Drusen volume is measured in each OCT scan between Bruch's membrane (BM) (red line) and the retinal pigment epithelium (RPE) (blue line). (A), (B), and (C) show an exemplary evaluation for a non-exudative AMD patient. [file 12967_2024_5909_MOESM1_ESM.jpg]

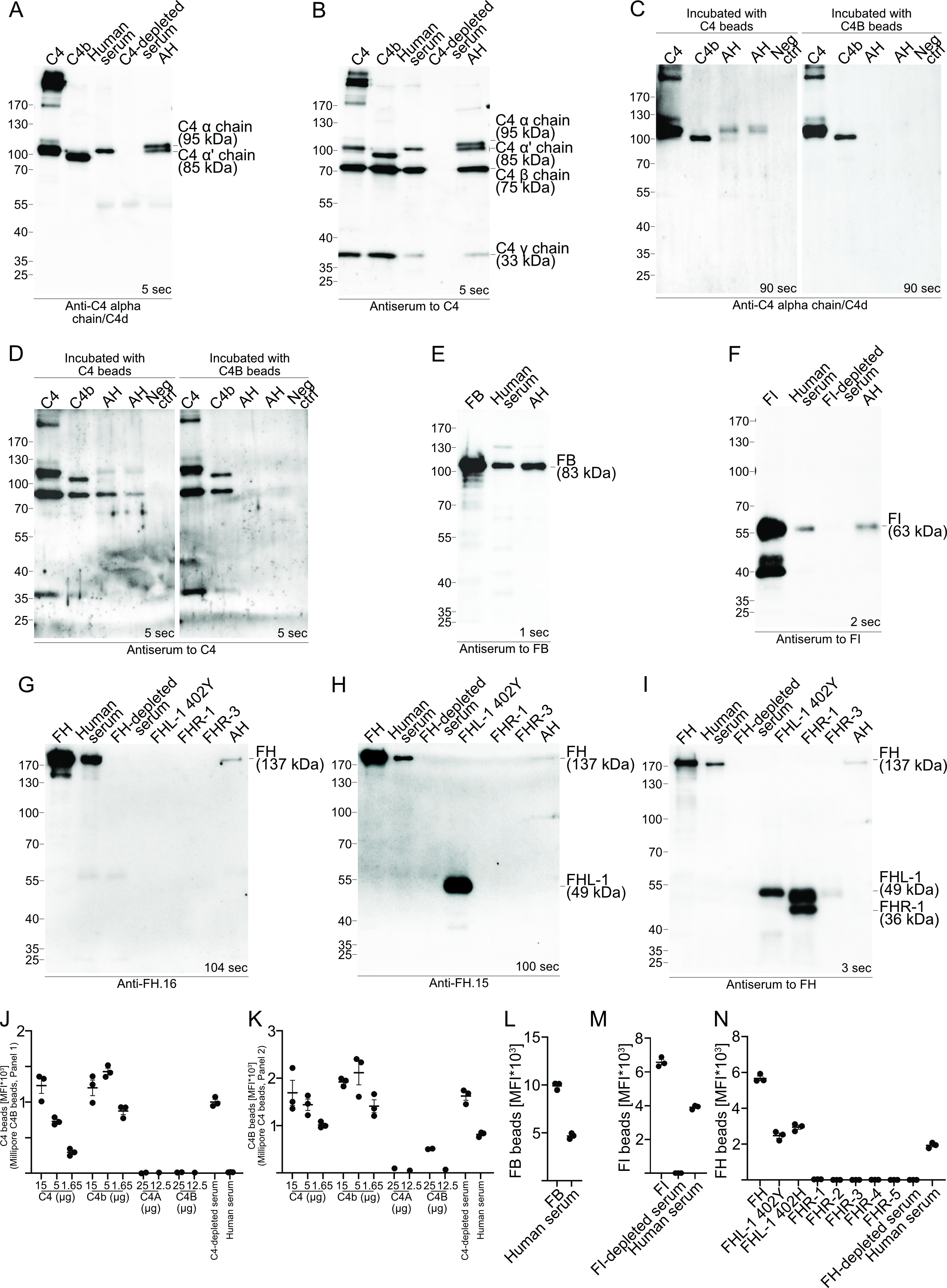

Supplement: Supplementary file 12 — Additional file 12. Complement protein detection by Western blot and relevant specificity controls for multiplex immunoassay. (A, B) The C4 α-chain and activated C4b α’-chain were identified in purified proteins, serum and AH. (B) Assessment of C4 subdomains (α-, β-, γ-chains) using polyclonal antiserum detected specific bands in purified proteins, serum, and AH. (C, D) Immune precipitation with C4B beads, but not C4 beads revealed the C4 α- and β-chain in AH. No signals were detectable in the negative control (neg ctrl). (E) Specific bands for FB were observable in purified FB, human serum, and AH. (F) Presence of FI was confirmed in purified FI, human serum, and AH, while it was absent in FI-depleted serum. (G) A specific antibody targeting complement control protein 19 of FH identified FH in a positive control, human serum, and AH. No signals were observed in FH-depleted serum or in purified FHL-1, FH-related (FHR)-1, and FHR-3 proteins. (H) Detection of FH and FH-associated proteins was performed with an antibody targeting complement control protein 5. It was exclusively found in purified FH and FHL-1, while no signals were observed for FHR-1 and FHR-3. (I) Analysis with polyclonal antiserum revealed a band for FH, FHL-1, and a distinct double band, consistent with reported signals for FHR-1. A band for FH was observable in AH. (J–N) Specificity controls were performed using the Luminex® xMAP® technology. (J, K) C4 beads as well as C4B beads identified purified C4, C4b, and human serum, while lower signals were observed in C4-depleted serum. Recombinant C4B was detect by C4-specific beads from panel 2. (L) FB was detected in purified FB and serum with FB-specific beads. (M) FI beads showed signals in purified FI and serum samples, but no signal in FI-depleted serum. (N) FH beads detected FH and both FHL-1 402Y and 402H variants, while no signals were observed for other FH-related components and FH-depleted serum. sec = exposure time in seconds. [file 12967_2024_5909_MOESM12_ESM.jpg]
