# Supplementary material for: Decreased complement 4 and interleukin-10 as biomarkers in aqueous humour for non-exudative age-related macular degeneration: a case control study
Source: J Transl Med. 2025 Mar 12;23:317. doi: 10.1186/s12967-024-05909-x (PMC11905602; doi:10.1186/s12967-024-05909-x)
Supplement: Supplementary file 4 — Additional file 4. Antibodies and proteins used in validation experiments (Additional file 12). Listed are concentrations/dilutions and references of the used antibodies, purified proteins, sera and the amount of AH used in validation experiments. Primary antibodies anti-FH.15 and anti-FH.16 were provided by Sanquin Research (Amsterdam, Netherlands) [96]. Recombinant FHL-1 402Y and 402H variants were produced by Józsi [97]. [file 12967_2024_5909_MOESM4_ESM.docx]

| **Sample/Antibody** | | **Concentration/**  **Dilution/ Amount** | **Reference** | **Catalog number** |
| --- | --- | --- | --- | --- |
| Primary antibody | anti-C4A alpha chain/C4d-A | 3 µg/mL | ProteinTech Group, Inc, Rose-mont, IL, USA | 22233-1-AP |
|  | antiserum to C4 | 8.2 µg/mL | Complement Technology, Inc, Tyler, TX, USA | A205 |
|  | anti-FH.15 | 5 µg/mL | provided by. R. Pouw | |
|  | anti-FH.16 |  |  |  |
|  | antiserum to FB | 1:500 | Quidel, San Diego, CA, USA | A311 |
|  | antiserum to FH | 4 µg/mL |  | A312 |
|  | antiserum to FI | 90 µg/mL |  | A313 |
| Secondary antibody | anti-goat horseradish peroxidase | 1:5000 | Dianova, Hamburg, Germany | 305-035-003 |
|  | anti-mouse horseradish peroxidase |  |  | 115-035-164 |
|  | anti-rabbit horseradish peroxidase |  |  | 111-035-003 |
| Purified proteins | C4 | Western blot/  immune precipitation: 100 ng  multiplex immunoassay: 100 ng/mL | Merck, Darmstadt, Germany | C8195 |
|  | C4b |  | Complement Technology, Inc. | A108 |
|  | FB |  |  | A408 |
|  | FH |  |  | A137 |
|  | FI |  |  | A138 |
|  | recombinant FHL-1 402Y variant |  | provided by M. Józsi | |
|  | recombinant FHL-1 402H variant |  |  |  |
|  | recombinant FHR-1 |  | Elabscience, Houston, TX, USA | PKSH033320 |
|  | recombinant FHR-2 |  |  | PKSH032275 |
|  | recombinant FHR-3 |  |  | PKSH033711 |
|  | recombinant FHR-4 |  |  | PKSH0333710 |
|  | recombinant FHR-5 |  |  | PKSH032274 |
|  | recombinant C4A*03 fragment | multiplex immunoassay:  25 µg | Inno-Train Diagnostik GmbH, Kronberg, Germany | 004010015 |
|  | recombinant C4B*03 fragment |  |  | 004010001 |
| Sera | C4-depleted serum | Western blot: 1:100  multiplex immunoassay: according to kit instructions | Complement Technology, Inc. | A308 |
|  | FH-depleted serum |  |  | A337 |
|  | FI-depleted serum |  |  | A338 |
|  | normal human serum |  |  | NHS |
| AH | AH sample | Western blot/  immune precipitation: 15 µL | presented study | - |
